# Optimization of Stripping Voltammetric Sensor by a Back Propagation Artificial Neural Network for the Accurate Determination of Pb(II) in the Presence of Cd(II)

**DOI:** 10.3390/s16091540

**Published:** 2016-09-21

**Authors:** Guo Zhao, Hui Wang, Gang Liu, Zhiqiang Wang

**Affiliations:** 1 Key Lab of Modern Precision Agriculture System Integration Research, Ministry of Education of China, China Agricultural University, Beijing 100083, China; 15264315915@163.com (G.Z.); wanghuilunwem@gmail.com (H.W.); 2 Key Lab of Agricultural Information Acquisition Technology, Ministry of Agricultural of China, China Agricultural University, Beijing 100083, China; 3 College of Computer Science and Technology, Shandong University of Technology, Zibo 255049, China; wzq@sdut.edu.cn

**Keywords:** square wave anodic stripping voltammetry, artificial neural network, bismuth film electrode, Pb(II), Cd(II)

## Abstract

An easy, but effective, method has been proposed to detect and quantify the Pb(II) in the presence of Cd(II) based on a Bi/glassy carbon electrode (Bi/GCE) with the combination of a back propagation artificial neural network (BP-ANN) and square wave anodic stripping voltammetry (SWASV) without further electrode modification. The effects of Cd(II) in different concentrations on stripping responses of Pb(II) was studied. The results indicate that the presence of Cd(II) will reduce the prediction precision of a direct calibration model. Therefore, a two-input and one-output BP-ANN was built for the optimization of a stripping voltammetric sensor, which considering the combined effects of Cd(II) and Pb(II) on the SWASV detection of Pb(II) and establishing the nonlinear relationship between the stripping peak currents of Pb(II) and Cd(II) and the concentration of Pb(II). The key parameters of the BP-ANN and the factors affecting the SWASV detection of Pb(II) were optimized. The prediction performance of direct calibration model and BP-ANN model were tested with regard to the mean absolute error (MAE), root mean square error (RMSE), average relative error (ARE), and correlation coefficient. The results proved that the BP-ANN model exhibited higher prediction accuracy than the direct calibration model. Finally, a real samples analysis was performed to determine trace Pb(II) in some soil specimens with satisfactory results.

## 1. Introduction

Lead is regarded as one of the most toxic heavy metals in the environment [1]. It can pose acute or chronic risks to ecosystem at very low concentrations because of their high toxicities, high stabilities and propagated accumulation through food chains [2]. This is to say, from plants grown in tainted agricultural land or meat from animals which have ingested plants grown in contaminated soil [3]. Therefore, it is necessary to establish a convenient, fast, and precise method for the detection of trace lead in soil to assess the potential risk of environmental pollution.

As an electrochemical technique, anodic stripping voltammetry (ASV) has been accepted to be one of the sensitive and convenient tools for the determination of trace metals [4]. In ASV [5] metal ions are electroplated onto the surface of an electrode and then electrically stripped off. The current which flows during the stripping process is proportional to the metal concentration, and the potential at which the stripping occurs corresponds to the type of metal involved in the reaction [6]. However, the presence of other heavy metal ions may influence the stripping currents of the target heavy metal ions [2,7,8,9], leading to low detection precision of direct calibration models. 

The study of chemically-modified electrodes (CMEs) is one of the active research areas in the ASV detection of heavy metals, which can effectively improve the detection performance of electrodes including effective preconcentration, selective reaction surface, good repeatability, and low background current over a wide potential range [2,10,11,12]. However, they suffer several limitations, i.e., expensive modification materials, and complex and time-consuming modification procedures, thus limiting any in situ and on-line analysis. Moreover, rigorous storage conditions are necessary to avoid damage and denaturation of modification materials on the electrode surface [13]. In order to improve the above drawbacks and detect Pb(II) at low cost and ease of use, Bi film-modified GCE was used for square wave anodic stripping voltammetry (SWASV) detection of Pb(II), owing to the low toxicity, the ability to form alloys with many heavy metal ions, simple preparation, and wide potential window [14,15,16,17].

In the past decades, there have been many papers reporting the use of CMEs in the ASV detection of Pb(II) [18,19,20,21,22]. Some research has considered the interference of Cd(II) on the SWASV detection of Pb(II), however, most of the reported works only considered the effect of Cd(II) on the SWASV detection of Pb(II) at a certain concentration instead of different concentrations of Cd(II). In this paper, the effects of Cd(II) in different concentrations on stripping voltammetric responses of Pb(II) was studied to analyze the relationship between the stripping peak currents of Pb(II) and Cd(II) and the concentrations of Pb(II). Moreover, a back propagation artificial neural network (BP-ANN) was used to discover, process, and interpret nonlinear relationships between the stripping peak currents of Pb(II) and Cd(II) and the concentrations of Pb(II), and create a simple and manageable mathematical model for Pb(II) detection. To the best our knowledge, very few reports on the combination of SWASV and BP-ANN could be found to detect Pb(II) in the presence of Cd(II). 

In this paper, a method based on the combination of SWASV and BP-ANN has been proposed for the optimization of Bi/GCE sensor to realize the accurate detection of Pb(II) in the presence of Cd(II). Consequently, the combination of SWASV and ANNs can be viewed as a source of many potential methods to detect and quantify many different kinds of heavy metal ions in various natural samples.

## 2. Experimental

### 2.1. Reagent and Apparatus

Stock solution of Bi(III), Pb(II) and Cd(II) (1000 mg/L) were obtained from National Standard Reference Materials Center of China (Beijing, China) and diluted as required. Acetate buffer solution (0.1 M) served as the supporting electrolyte for the detection of Pb(II). All other chemicals were of analytical grade and used without further purification. Millipore-Q (18.2 MΩ) (Beijing Science and Technology Development Co., Ltd., Beijing, China) water was used for all experiments.

Square wave anodic stripping voltammetry was performed on a CHI660D electrochemical workstation (Shanghai CH Instruments, Shanghai, China). The three-electrode system consisted of a Bi/glassy carbon working electrode (Φ = 3 mm), an Ag/AgCl reference electrode, and a counter electrode made of platinum wire. All electrochemical measurements were carried out in a 25 mL cell. A magnetic stirrer was used to stir the test solutions during the deposition steps.

### 2.2. Preparation of Bi/Glassy Carbon Electrode

Prior to the electrodeposition of bismuth film, the GCE was first polished with 0.05 μm alumina slurry, sequentially, and then washed ultrasonically with HNO_3_–H_2_O (1:1 by volume), absolute ethanol, and water, respectively, and finally dried in N_2_ atmosphere. A pretreated GCE was immersed into 0.1 M acetate buffer solution containing 600 μg/L Bi(III), the pH of which was 5.0, and a bismuth film was electrodeposited onto the GCE by potentiostatic treatment at −1.4 V (versus Ag/AgCl) for 140 s to form Bi/GCE.

### 2.3. Pb(II) Detection by SWASV in the Presence of Cd(II)

SWASV was used for the Pb(II) detection under optimized conditions, with an in situ deposition of bismuth film. The Bi/GCE, Ag/AgCl, and platinum wire electrodes were immersed into an electrochemical cell, which contained 20 mL 0.1 mol/L acetate buffer (pH 5.0), 600 μg/L Bi(III), and different concentrations of Pb(II) and Cd(II) ranging from 1 to 110 μg/L. In the deposition process, the working electrode was provided with −1.2 V for 140 s, under stirring conditions. After an equilibration period of 10 s, the square wave anodic stripping voltammogram was recorded from −1.2 to 0.2 V, and the solution was not stirred in these two steps. The potential step, square wave amplitude, and frequency were 5 mV, 25 mV, and 25 Hz, respectively. Prior to the next determination, the modified electrode was activated for 120 s at 0.31 V in a pH 5.0 acetate buffer to remove the residual metals and bismuth film from the surface of the working electrode under stirring conditions. All of the experiments were carried out at room temperature.

### 2.4. Artificial Neural Network Modelling

Artificial neural networks (ANNs) are inspired by biological neural systems. The weighted sum of inputs arriving at each neuron is passed through an activation function to generate an output signal [23,24]. ANNs have good flexibility that does not need a rigid mathematical model and the calibration parameters are able to be determined using data through a learning step [25]. BP-ANN, as one of the most employed ANN methods, has been selected for this study due to its wide application to discover and interpret nonlinear relationships present in databases [26,27,28]. The BP-ANN used, which is trained with data from the SWASV spectrum, is supervised because it requires target data (known Pb(II) concentrations, in this case) for its correct training and optimization [29].

In this paper, the accurate detection of Pb(II) in the presence of Cd(II) has been attempted based on the combination of the BP-ANN and SWASV with a Bi/GCE. BP-ANNs are formed by three types of layers, which are input, hidden, and output. The input layer is formed by nodes, and these are used to select the number of independent variables that are used in the estimative tool [30]. Hidden and output layers are both composed of neurons, which are the actual calculation centers of the BP-ANN. The hidden neuron number (HNN) should be adequately optimized to find the network topology that provides the best statistical results. The correct definition of the HNN is fundamental because a low HNN may negatively affect the learning ability of the BP-ANN. In contrast, when the HNN is too high, the resulting ANN may be over-fit toward the employed dataset [31]. Finally, the output neurons are selected depending on the dependent variables that are trying to be estimated [32].

**Figure 1:**
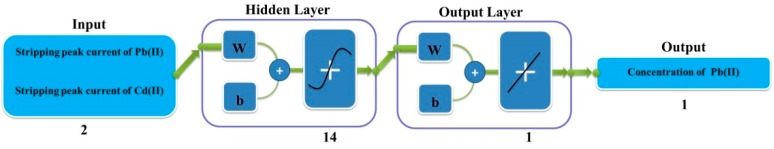
Schematic of an ANN structure to predict the concentration of Pb(II).

In this study, a three-layer ANN was used to predict the non-linear relationship between the Pb(II) concentration and the stripping peak currents of Pb(II) and Cd(II). The inputs were the stripping peak currents of Pb(II) and Cd(II), respectively, and the concentration of Pb(II) was the output of the ANN model, as shown in Figure 1.

BP-ANN-related parameters have been selected, while others have been optimized to find the best possible model for the estimation of the concentration of Pb(II) in the presence of Cd(II), which is evaluated through its mean absolute error (MAE; Equation (1)), root mean square error (RMSE; Equation (2)), average relative error (ARE; Equation (3)), and *R*^2^ correlation coefficient (Equation (4)). The selected parameters were the hidden neuron number, transfer function, and training function. All ANN-related calculations and simulations have been carried out utilizing MATLAB R2012b (The Mathworks, Inc., Natick, MA, USA).

(1) $$\mathrm{MAE}=\frac{1}{n}\overset{n}{\underset{i=1}{\sum }}\left|X_{pi}-X_{ai}\right|$$

(2) $$\mathrm{RMSE}=\sqrt{\frac{\sum_{i=1}^{n}{\left(X_{pi}-X_{ai}\right)}^{2}}{n}}$$

(3) $$\mathrm{ARE}=\frac{1}{n}\overset{n}{\underset{i=1}{\sum }}\frac{\left|X_{pi}-X_{ai}\right|}{X_{ai}}$$

(4) $$R^{2}=\frac{n\sum_{i=1}^{n}X_{pi}X_{ai}-\left(\sum_{i=1}^{n}X_{pi}\right)\left(\sum_{i=1}^{n}X_{ai}\right)}{\left(n(\sum_{i=1}^{n}{X_{pi}}^{2})-{\left(\sum_{i=1}^{n}X_{pi}\right)}^{2}\right)\left(n\left(\sum_{i=1}^{n}{X_{ai}}^{2}\right)-{\left(\sum_{i=1}^{n}X_{ai}\right)}^{2}\right)}$$

where *n*, *X_pi_*, and *X_ai_* are the total number of predictions, predicted, and actual values (experimental values), respectively.

### 2.5. Soil Samples Preparation

Soil samples were collected from a cultivated land in China. The extraction process was performed according to the published literature [8,33]. Briefly, soil samples were dried in an oven for about 2 h. Then, the samples were ground by a mortar and sieved by a 200 μm sieve. A portion (1 g) of soil was transferred to an extraction bottle in which 40 mL of 0.11 M acetic acid was added. The mixed sample was shaken on an end-over-end shaker for 16 h at room temperature. Then, the mixture was centrifuged for phase separation and the aqueous phase was filtered through a membrane of 0.2 μm pore size. Before the measurement, the pH of the extract solutions was adjusted to 5.0 by addition of NaOH solution (0.11 M).

## 3. Result and Discussion

### 3.1. Optimization of Experimental Conditions

**Figure 2:**
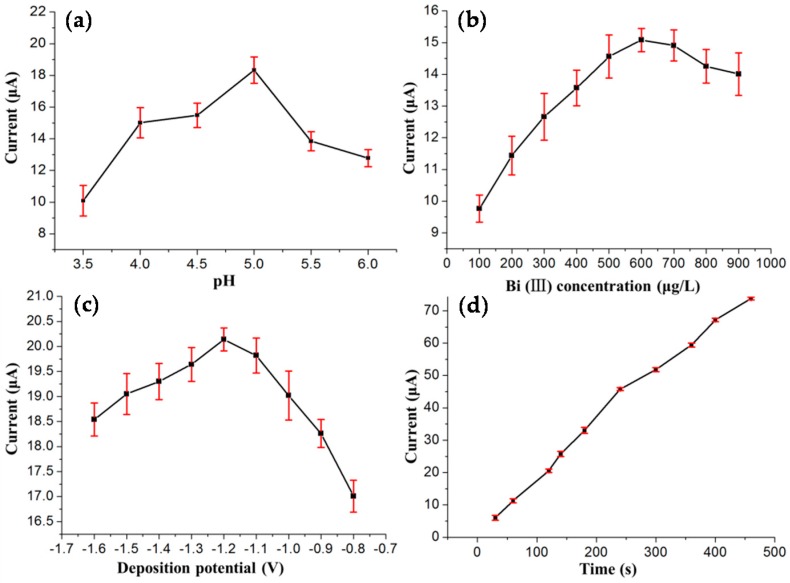
Effects of (**a**) pH value; (**b**) Bi(III) concentration (**c**) deposition potential; and (**d**) deposition time on the stripping peak currents of 50 μg/L Pb(II) in the presence of 20 μg/L Cd(II).

The effect of the pH value of the supporting electrolyte on the stripping peak current of Pb(II) was studied. Figure 2a shows the effect of pH on the stripping response of Pb(II) in the range from 3.5–6. The maximum peak current appeared at pH 5.0. The reason for this observation may be because, at high acidity, the reduction of H^+^ to H_2_ occurred on the working electrode, which blocked the reduction of the target metals onto the electrode surface, and a too high pH value can result in hydrolysis of Cd(II) and Pb(II). Finally, the pH 5.0 was chosen for the following experiments. Figure 2b demonstrates the effect of the Bi(III) concentration on the stripping response of Pb(II). The peak currents increased with the increasing concentration of Bi(III) from 100 to 600 μg/L, and then decreased as the concentration of Bi(III) exceeded 600 μg/L. This phenomenon might be attributed to the formation of a thick bismuth film on the electrode surface, which was not favorable for target heavy metal ions diffusing out. Consequently, we chose 600 μg/L as the optimal Bi(III) concentration. The effect of the deposition potential on the stripping response of Pb(II) over the potential range of −0.8 to −1.6 V after 140 s accumulation was explored and shown in Figure 2c. The highest stripping currents were obtained at −1.2 V for Pb(II), since more positive potentials may be inefficient for the reduction of Pb(II), but more negative potentials could cause hydrogen evolution and interfere with the Pb(II) detection [34]. For further measurements, a value of −1.2 V was chosen as the accumulation potential. As the deposition time plays an important role in improving the sensitivity of SWASV measurement, a series of accumulation times from 30–460 s were investigated to ensure the relationship between deposition time and peak current responses. Figure 2d shows the effect of the deposition time on the stripping peak currents of Pb(II); as can be seen, the stripping peak currents of Pb(II) were almost linearly proportional to deposition time up to 460 s. In order to achieve high sensitivity within a relatively short analysis time, a deposition time of 140 s was chosen. The standard deviations obtained by repeated measurements of five times, in the form of error bars, can be seen in Figure 2. The standard deviations of these four parameters were distributed between 0.23 and 0.97.

### 3.2. Electrochemical Characteristic of the Bi/GCE

**Figure 3:**
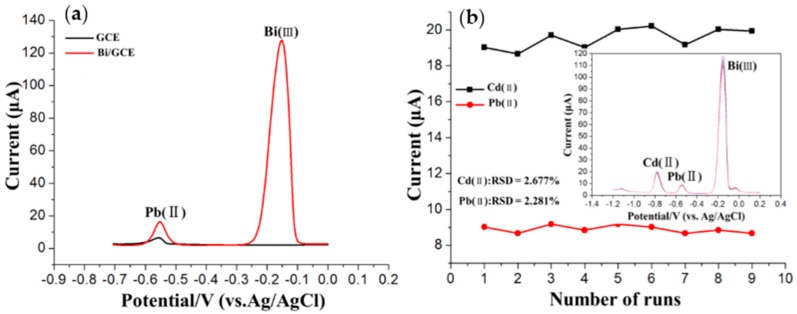
(**a**) Square wave anodic stripping voltammograms of 35 μg/L Pb(II) in 0.1 M acetate buffer solution (pH 5.0) on GCE and Bi/GCE. Deposition time: 140 s; deposition potential: −1.2 V; concentration of Bi(III): 600 μg/L; (**b**) Stripping current measurements of 25 μg/L Cd(II) and Pb(II) on Bi/GCE in 0.1 M acetate buffer solution (pH 5.0). The insets correspond to data collected from every SWASV response over nine repetitions. RSD: relative standard deviation.

The high stripping peak current is the prerequisite to obtain a high detection precision. Figure 3a compares the square wave stripping peak currents of 35 μg/L Pb(II) at the GCE and Bi/GCE. As shown, the stripping peak current of Pb(II) at the GCE were unobvious and weak. In contrast, the Bi/GCE exhibited higher stripping peak currents toward Pb(II). The reason might be that Bi can “alloy” with heavy metals which make them more easily reduced. The stability and reproducibility of the Bi/GCE was tested via nine repetitive measurements of 25 μg/L Pb(II) and Cd(II) in 0.1 M acetate buffer solution (pH 5.0), as shown in Figure 3b. The stripping peak currents of the Pb(II) and Cd(II) on the Bi/GCE were reproducible, with relative standard deviations (RSDs) of 2.677% for Cd(II) and 2.281% for Pb(II), respectively. The results show that the Bi/GCE exhibits excellent stability and reproducibility in repeated stripping measurements of these HMs under the optimum experimental conditions, which provided stable and accurate modeling data for the ANN model.

### 3.3. Effects of Cd(II) on the SWASV Detection of Pb(II)

**Figure 4:**
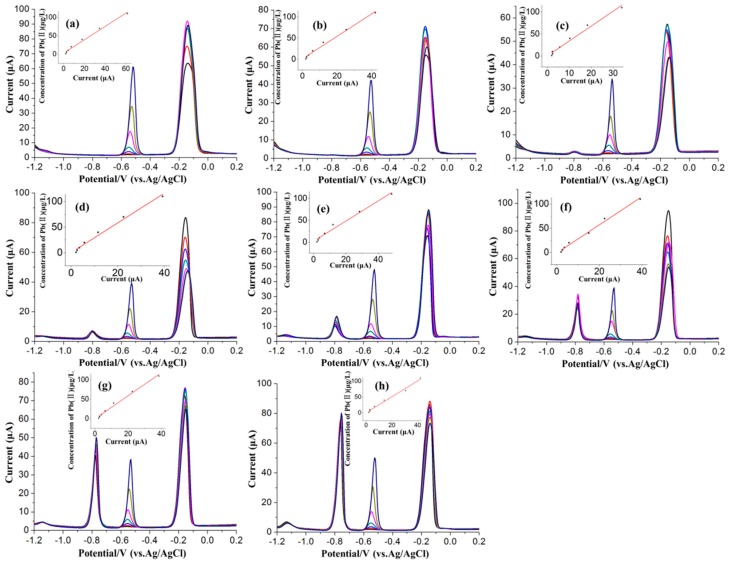
Voltamogrames for Pb(II) ranged from 1.0 to 110 μg/L in different concentrations of Cd(II) ranged from 0 to 110 μg/L: (**a**) 0 μg/L; (**b**) 1 μg/L; (**c**) 5 μg/L; (**d**) 10 μg/L; (**e**) 20 μg/L; (**f**) 40 μg/L; (**g**) 70 μg/L; (**h**) 110 μg/L. Deposition time: 140 s. Deposition potential: −1.2V. Concentration of Bi(III): 600 μg/L.

**Figure 5:**
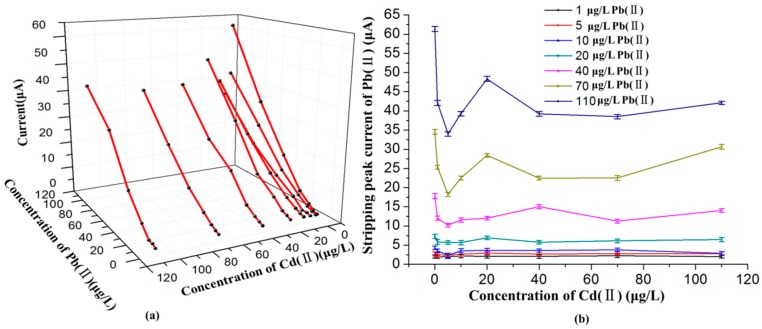
(**a**) The effects of different concentrations of Cd(II) on the fitting curve of Pb(II); (**b**) The effects of different concentrations of Cd(II) on the stripping peak currents of Pb(II).

In order to investigate the effects of Cd(II) in different concentrations on the SWASV detection of Pb(II) based on Bi/GCE, additional studies were performed on the stripping processes of these two metal ions in their mixtures. Several binary mixtures of two species were prepared, in which the concentration of Pb(II) changed from 1.0 to 110 μg/L, whereas the concentration of Cd(II) remained constant at a specified value in the range of 0 to 110 μg/L, as shown in Figure 4. The SWASV voltammograms of these mixed solutions with different combinations of Pb(II) and Cd(II) were recorded under the optimum conditions. The obtained results in Figure 4 show that the stripping peak current of Pb(II) was linear to the concentration of Pb(II) approximately in the range of 1.0 to 110 μg/L, while the stripping responses of Pb(II) were practically affected by the Cd(II) in different concentrations. As can be seen from the Figure 5a, the calibration curve of Pb(II) changed obviously with the change of the Cd(II) concentration compared with the absence of Cd(II). When the concentration of Cd(II) was close to about 5 μg/L, the interference of the Cd(II) to the stripping responses of Pb(II) was the most significant. When the concentration of Cd(II) was about 40 μg/L, the interference of the Cd(II) to the stripping peak currents of Pb(II) still existed, but remained relatively stable, as shown in Figure 5b. The standard deviations obtained by repeated measurements of five times, in the form of error bars, also can be seen in the Figure 5b. The standard deviations of the stripping peak current were distributed between 0.42 and 0.66. The interference of the Cd(II) to the responses of Pb(II) could be explained as follows. Due to the close similarity in electrochemical behavior, both Cd(II) and Pb(II) could form binary [34]. Moreover, Cd and Pb could form multicomponent alloys with Bi, which may block the reduction of the Pb(II) onto the electrode surface at the accumulation step and affect the normal stripping of Pb at stripping step.

The standard errors of the linear regression parameters, including slope and intercept, have been presented in Table 1. Moreover, the confidence level of and the significance of linear regression equations have been also presented in Table 1. The values of “Prob > F” were all less than 0.01, which indicated the high significance of the linear regression equations. These experimental results could be explained as follows. In ASV, metal ions are electroplated onto the surface of an electrode and then electrically stripped off. The current which flows during the stripping process has a good linear relationship with the concentration of target heavy metal ions. Due to the good repeatability and stability of Bi film modified glass carbon electrode used for heavy metals detection (cf. Section 3.2), satisfactory experimental errors could be expected. Although good linearity and significance of linear regression equations could be obtained, the linear regression equations used for the Pb(II) detection were different in the presence of Cd(II) of different concentration. In the actual detection, we did not know the concentration of Cd(II) in actual samples, therefore, we could not choose the most suitable linear equation for the detection of Pb(II) in this case. 

In order to verify the difference of different linear regression equations for the detection of Pb(II), as an example, the detection of 110 μg/L Pb(II) in the presence of 5 μg/L Cd(II) was carried out by these equations. Prediction results of different calibration linear equations can be seen in Table 2 with measurements repeated five times, which indicated the difference of prediction performance of the different linear equations. It can be concluded that the presence of Cd(II) will lead to low detection precision of Pb(II) by the direct calibration model. The presence of Cd(II) in different concentrations will reduce the detection accuracy of target Pb(II) by the directed calibration equation to different degrees.

### 3.4. Proposed BP-ANN Model for the Detection of Pb(II)

#### 3.4.1. Parameters Selection and Optimization

The developed two-input and one-output BP-ANN model (cf. Figure 1) for the prediction of Pb(II) is made of three layers, each of the layers is interconnected by processing elements known as neurons. The number of hidden neurons determines the model complexity of an ANN [35]. Training a BP-ANN is an essential step to ensure prediction accuracy of the ANN model. This step entails utilization of a training dataset to train the network [15]. Moreover, a testing dataset was used for verifying the prediction precision of the proposed BP-ANN model.

In order to eliminate the influence of magnitude and improve network convergence performance, sample data, including the training dataset and the testing dataset, have been normalized. The input and output variables were normalized based on Equation (5), as follows:

(5) $$X_{k}^{\prime }=\frac{\left(x_{max}^{\prime }-x_{min}^{\prime }\right)\left(x_{k}-x_{min}\right)}{\left(x_{max}-x_{min}\right)}+x_{min}^{\prime }$$

where *x_max_* represents the maximum value of the variable and *x_min_* represents its minimum value. $x_{max}^{\prime }$ was set as 1 and $x_{min}^{\prime }$ was set as −1. After preprocessing, the range of variables was normalized as [−1, 1].

In the training process, the simulation efficiency of the network could be affected by the training parameters, especially the number of neurons in the hidden layer, transfer function, and training function [25,36,37]. Therefore, figuring out the optimal combination of these parameters for an ANN was an important task. In this paper, simulations were carried out for different numbers of neurons, different transfer functions, and different training functions so that the best prediction scheme for the neural network can be determined. The number of neurons in the hidden layer was determined based on empirical formulas, as shown in Equation (6) [38]. In this equation, *n_h_* is the number of hidden layer neurons, *n_i_* is the number of input layer neurons, *n_o_* is the number of output layer neurons, and *l* is a constant that varies from 1 to 10.

(6) $$n_{h}=\sqrt{n_{i}+n_{o}}+l$$

**Figure 6:**
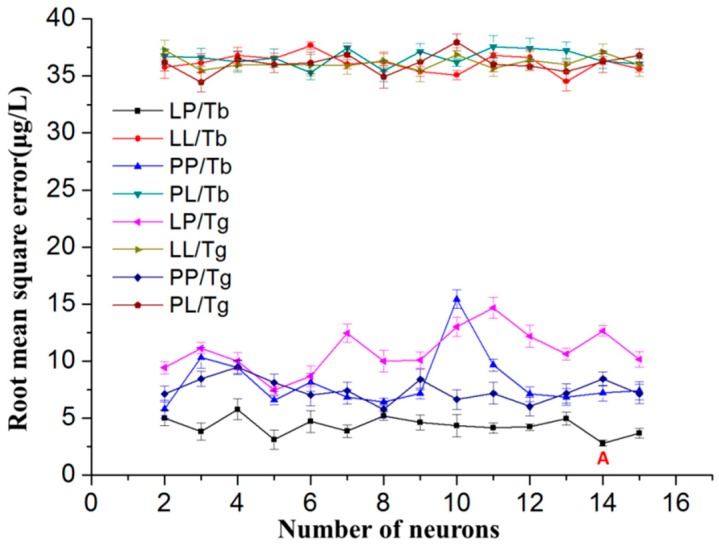
Selection and optimization of the ANN model. The transfer functions and training functions: Logsig and Pureline (LP), Logsig and Logsig (LL), Pureline and Pureline (PP), Pureline and Logsig (PL), Trainbr (Tb), and Traingdx (Tg).

According to the equation, the number of neurons in the hidden layer was selected from 2–15. The standard deviations obtained by measurements repeated five times, in the form of error bars, can also be seen in the Figure 6. The standard deviations of RMSE were distributed between 0.24 and 0.98. Different transfer functions and training functions, such as purelin and logsig, Trainbr, and Traingdx, were tested in order to get the best modeling network. For example, on the basis of data in Figure 6, the minimum RMSE corresponds to position “A”, which shows the learning functions Logsig and Pureline for the hidden layer and output layer as optimums for manganese, respectively. In the optimization of the number of neurons, the number of neurons with smaller error is needed. Taking into account the special needs of trace detection of heavy metals (μg/L level), the ANN model was optimized to get a high prediction accuracy as far as possible. Compared with the modeling network with five neurons, the modeling network with 14 neurons surely increased the complexity of the modeling network; however, the increase of nine neurons would not significantly affect the operating efficiency of the program and the actual detection efficiency. Taking into account both the operating efficiency of the program and the actual detection efficiency of the ANN model, the modeling network with 14 neurons was used.

#### 3.4.2. Establishment and Validation of the BP-ANN Model

By using the optimal parameter combination above, 37 sets of experimental data (training dataset) were used to train the ANN model, and 12 sets of experimental data were used as the testing dataset. Tables S1 and S2 (Supplementary Materials) were the prediction results of the training dataset and the testing dataset, respectively. Statistical parameters, such as MAE, RMSE, ARE, and correlation coefficient of determination depicted in Equations (1)–(4) were used to evaluate the fitness and the prediction accuracy of the ANN model. The statistical analysis showing the comparison results of the training dataset and testing dataset are depicted in Table 3. The reasonable MAE, RMSE, and *R*^2^ for both training dataset and testing dataset indicate the prediction accuracy of the BP-ANN model for the prediction of Pb(II).

**Figure 7:**
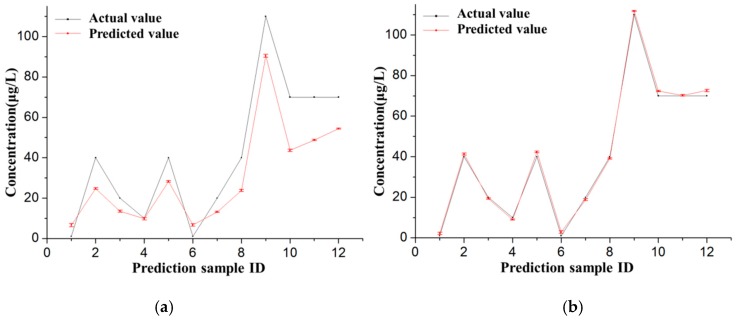
The comparison of prediction results between (**a**) the direct calibration model and (**b**) the ANN model.

**Figure 8:**
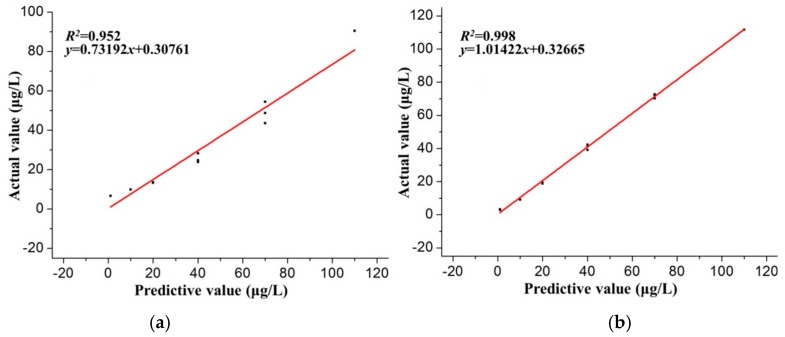
Linear regression analysis of the prediction results obtained from (**a**) the direct calibration model and (**b**) the ANN model.

The predicted outputs (concentration of Pb(II)) of the testing dataset from the well-trained BP-ANN model were compared with the actual values, as shown in Figure 7. The standard deviations were obtained in the form of error bars by repeating measurements five times. The standard deviations of concentration were distributed between 0.27 and 0.63 for the ANN model, and between 0.32 and 0.79 for the direct calibration model. This was to validate the prediction accuracy of the trained BP-ANN model. The MAE, RMSE, ARE, and *R*^2^ of the direct calibration model for the prediction of the testing dataset was compared with the ANN model to further demonstrate the applicability of the developed BP-ANN model. The linear regression analysis shown in Figure 8 indicates that there is a strong correlation (*R*^2^ = 0.998) between the predicted values from the BP-ANN model and the actual values compared with the direct calibration model (*R*^2^ = 0.952).

The comparison of statistical parameters between the direct calibration model and the ANN model are depicted in Table 4. The MAE, RMSE, and ARE of the BP-ANN model at optimum conditions were estimated to be 1.52 μg/L, 1.69 μg/L, and 11.24 μg/L. The corresponding statistical parameters of the direct calibration model were 12.56 μg/L, 14.60 μg/L, and 28.878 μg/L, respectively. Further statistical analysis shows that the BP-ANN model has better prediction precision compared with the direct calibration model. According to [39], the better prediction performance of the BP-ANN model compared to the direct calibration model may be due to the tendency for the ANNs to approximate the non-linearity of the system. The poor prediction performance of the direct calibration model may be attributed to the direct calibration model having a low predictive power for the Pb(II) in, and the presence of, Cd(II).

Furthermore, the repeatability of the proposed method was evaluated by repeating the determination of 50 μg/L Pb(II) in the presence of 20 μg/L Cd(II) using six modified electrodes in the same batch. The relative standard deviation (RSD) was 3.8%, which suggested acceptable repeatability and precision.

### 3.5. Application to Real Sample Analysis

The developed method was applied for the determination of Pb(II) in soil samples to evaluate the application performance. The measurement was performed by standard addition method. To reduce the interference of Cu(II) and carry out an accurate prediction of Pb(II), the soil samples were treated by the addition of ferrocyanide ions before the determination of Pb(II) according to our previous studies [20]. Additionally, different concentrations of standard solutions were added to the sample solutions and the recovery was subsequently evaluated. Table 5 shows the results obtained for the analysis of the real samples by the proposed method. The results show satisfactory recovery results with an average recovery of 98.42%, confirming the availability of the developed method.

## 4. Conclusions

A BP-ANN have been applied to the optimization of a stripping voltammetric sensor and the analysis of the SWASV spectrum for the prediction of Pb(II) within the range of 1–110 μg/L. The key contributions of this paper not only focus on the mathematical modeling itself, but also consider Cd(II), which affects the SWASV detection of Pb(II), by integrating potential causative variables into the models. To the best of our knowledge, this is the first report of the combination of SWASV and the BP-ANN model using Bi/GCE to predict the concentration of Pb(II) in the presence of Cd(II). The effects of Cd(II) in different concentrations on the stripping voltammetric responses of Pb(II) were studied. The reasonable MAE, RMSE, ARE, and *R*^2^ for both the training dataset and the testing dataset indicate the accuracy of the proposed BP-ANN model for the prediction of Pb(II). The comparison of statistical parameters between the direct calibration model and the BP-ANN model shows that the BP-ANN model has better prediction precision than the direct calibration model. The obtained prediction performance of the proposed method for the real samples as an average recovery percentage was 98.42%, confirming the availability of the developed method. The method proposed in this paper implies that the combination of ASV and machine learning algorithms, such as ANNs, can be a new way to accurately estimate the concentration of heavy metals for environmental control, food safety supervision, and many other fields and applications.

## Figures and Tables

**Table 1:** Calibration equations of Pb(II) in different concentrations of Cd(II).

Concentration of Cd(II) (μg/L)	Calibration Linear Equation of Pb(II)				Adjust R-Square	Prob > F	Confidence Level (%)
	Slope		Intercept				
	Value	Standard Error	Value	Standard Error			
0	1.81136	0.08873	2.8841	2.45566	0.98577	5.22 × 10^−6^	95
1	2.6529	0.12407	1.1957	2.39892	0.98702	4.15 × 10^−6^	95
5	3.38167	0.20087	0.3585	3.07612	0.9792	1.35 × 10^−5^	95
10	2.8954	0.14708	0.4616	2.63022	0.98471	6.25 × 10^−6^	95
20	2.30259	0.13952	2.2375	3.05889	0.97837	1.49 × 10^−5^	95
40	2.90499	0.12191	−1.1498	2.22284	0.98953	2.42 × 10^−6^	95
70	2.97084	0.1474	−0.4567	2.60129	0.98541	5.56 × 10^−6^	95
110	2.5196	0.13286	0.2467	2.73749	0.98355	7.51 × 10^−6^	95

**Table 2:** Prediction results of 110 μg/L Pb(II) in the presence of 5 μg/L Cd(II) by linear equations.

Concentration of Cd(II) (μg/L)	Calibration Linear Equation of Pb(II)	Mean Absolute Error (μg/L)	Average Relative Error (%)
0	Y = 1.81136 X + 2.88406	45.55	41.41
1	Y = 2.6529 X + 1.19574	18.63	16.94
5	Y = 3.38167 X + 0.35853	5.30	4.82
10	Y = 2.8954 X + 0.46164	11.12	10.11
20	Y = 2.30259 X + 2.23745	29.5	26.82
40	Y = 2.90499 X − 1.14984	12.41	11.28
70	Y = 2.97084 X − 0.45666	9.48	8.62
110	Y = 2.5196 X + 0.24673	24.11	21.92

**Table 3:** The statistical parameters of training dataset and testing dataset.

Data Set	MAE (μg/L)	RMSE (μg/L)	ARE (%)	*R*^2^
**Training Dataset**	0.89	1.31	11.24	0.999
**Testing Dataset**	1.52	1.69	28.88	0.998

**Table 4:** The statistical parameters of the direct calibration model and BP-ANN model.

Prediction Method	MAE (μg/L)	RMSE (μg/L)	ARE (%)	*R*^2^
**Direct Calibration**	12.56	14.6	117.61	0.952
**Artificial Neural Network**	1.52	1.69	28.88	0.998

**Table 5:** Results for the detection of Pb(II) in several soil sample extracts.

Sample No.	Added (μg/L)	Found (μg/L) ^a^	RSD	Recovery (%)
**1**	-	3.73	3.13	-
	4	7.59	2.18	96.5
	8	11.62	2.49	98.63
**2**	-	4.55	3.32	-
	15	19.27	2.51	98.13
	30	34.21	1.23	98.87
**3**	-	6.79	1.57	-
	45	51.18	3.41	98.64
	90	96.54	1.22	99.72

^a^ SWASV measurements were repeated five times (n = 5).

## Supplementary Materials

The following are available online at http://www.mdpi.com/1424-8220/16/9/1540/s1, Table S1: Experimental design and results of the training dataset, Table S2: Experimental design and results of the testing dataset.
