# Supplementary material for: Optimization of Stripping Voltammetric Sensor by a Back Propagation Artificial Neural Network for the Accurate Determination of Pb(II) in the Presence of Cd(II)
Source: Sensors (Basel). 2016 Sep 21;16(9):1540. doi: 10.3390/s16091540 (PMC5038813; doi:10.3390/s16091540)
Supplement: Supplementary file 1 [file sensors-16-01540-s001.pdf]

# Supplementary Materials: Optimization of Stripping Voltammetric Sensor by a Back Propagation Artificial Neural Network for the Accurate Determination of Pb(II) in the Presence of Cd(II)

**Guo Zhao, Hui Wang, Gang Liu and Zhiqiang Wang**

**Table S1.** Experimental design and results of the training dataset.

| No. | Stripping Peak Current | | Concentration (μg/L) | | Artificial Neural Network Modelling (μg/L) | Absolute Error (μg/L) | Relative Error (%) |
|---|---|---|---|---|---|---|---|
| | Cd(II) | Pb(II) | Cd(II) | Pb(II) | Pb(II) | | |
| 1 | 2.688 | 2.44 | 5 | 5 | 5.317 | 0.317 | 6.34 |
| 2 | 6.51 | 39.27 | 10 | 110 | 107.197 | 2.803 | 2.5482 |
| 3 | 11.53 | 2.134 | 20 | 1 | 1.138 | 0.138 | 13.80 |
| 4 | 40.64 | 2.234 | 70 | 1 | 1.13 | 0.130 | 130 |
| 5 | 7.246 | 2.143 | 10 | 1 | 2.195 | 1.195 | 119.50 |
| 6 | 24.96 | 5.785 | 40 | 20 | 19.522 | 0.478 | 2.390 |
| 7 | 2.114 | 3.556 | 1 | 10 | 10.075 | 0.075 | 0.750 |
| 8 | 43.69 | 3.81 | 70 | 10 | 10.519 | 0.519 | 5.190 |
| 9 | 2.954 | 10.23 | 5 | 40 | 39.067 | 0.933 | 2.333 |
| 10 | 2.722 | 18.28 | 5 | 70 | 69.469 | 0.531 | 0.759 |
| 11 | 78.41 | 30.68 | 110 | 70 | 69.911 | 0.089 | 0.127 |
| 12 | 1.953 | 1.964 | 1 | 1 | 1.023 | 0.023 | 2.30 |
| 13 | 43.24 | 2.819 | 70 | 5 | 5.341 | 0.341 | 6.820 |
| 14 | 44.86 | 6.124 | 70 | 20 | 21.625 | 1.625 | 8.125 |
| 15 | 3.116 | 5.664 | 5 | 20 | 19.276 | 0.724 | 3.620 |
| 16 | 12.75 | 2.921 | 20 | 5 | 5.174 | 0.174 | 3.480 |
| 17 | 24.66 | 3.595 | 40 | 10 | 9.669 | 0.331 | 3.310 |
| 18 | 1.953 | 42.12 | 1 | 110 | 110.695 | 0.695 | 0.632 |
| 19 | 12.73 | 12.04 | 20 | 40 | 39.668 | 0.332 | 0.830 |
| 20 | 2.685 | 33.99 | 5 | 110 | 109.472 | 0.528 | 0.480 |
| 21 | 28.24 | 39.21 | 40 | 110 | 110.252 | 0.252 | 0.229 |
| 22 | 2.885 | 3.339 | 5 | 10 | 9.94 | 0.060 | 0.60 |
| 23 | 44.84 | 11.26 | 70 | 40 | 35.037 | 4.963 | 12.408 |
| 24 | 50.53 | 38.5 | 70 | 110 | 110.759 | 0.759 | 0.690 |
| 25 | 6.85 | 3.542 | 10 | 10 | 9.595 | 0.405 | 4.050 |
| 26 | 76.56 | 2.778 | 110 | 5 | 6.879 | 1.879 | 37.58 |
| 27 | 46.91 | 22.5 | 70 | 70 | 68.59 | 1.410 | 2.014 |
| 28 | 10.31 | 3.589 | 20 | 10 | 9.537 | 0.463 | 4.630 |
| 29 | 25.19 | 2.701 | 40 | 5 | 6.249 | 1.249 | 24.980 |

**Table S1.** *Cont.*

| No. | Stripping Peak Current | | Concentration (μg/L) | | Artificial Neural Network Modelling (μg/L) | Absolute Error (μg/L) | Relative Error (%) |
|---|---|---|---|---|---|---|---|
| | Cd(II) | Pb(II) | Cd(II) | Pb(II) | Pb(II) | | |
| 30 | 1.968 | 2.468 | 1 | 5 | 5.467 | 0.467 | 9.340 |
| 31 | 2.595 | 2.017 | 5 | 1 | 1.867 | 0.867 | 86.70 |
| 32 | 6.991 | 2.599 | 10 | 5 | 5.659 | 0.659 | 13.180 |
| 33 | 34.59 | 15.03 | 40 | 40 | 42.43 | 2.430 | 6.075 |
| 34 | 73.74 | 6.451 | 110 | 20 | 19.512 | 0.488 | 2.440 |
| 35 | 6.378 | 22.51 | 10 | 70 | 71.327 | 1.327 | 1.896 |
| 36 | 14.12 | 6.913 | 20 | 20 | 22.37 | 2.370 | 11.850 |
| 37 | 73.59 | 42.1 | 110 | 110 | 109.034 | 0.966 | 0.878 |

**Table S2.** Experimental design and results of the testing dataset.

| No. | Stripping Peak Current | | Concentration (μg/L) | | Artificial Neural Network Modelling (μg/L) | Absolute Error (μg/L) | Relative Error (%) |
|---|---|---|---|---|---|---|---|
| | Cd(II) | Pb(II) | Cd(II) | Pb(II) | Pb(II) | | |
| 1 | 74.03 | 2.057 | 110 | 1 | 2.165 | 1.165 | 116.5 |
| 2 | 2.032 | 12.05 | 1 | 40 | 41.327 | 1.327 | 3.318 |
| 3 | 2.074 | 5.875 | 1 | 20 | 18.358 | 1.642 | 8.210 |
| 4 | 74 | 3.832 | 110 | 10 | 9.012 | 0.988 | 9.880 |
| 5 | 75.16 | 14.02 | 110 | 40 | 42.337 | 2.337 | 5.843 |
| 6 | 32.52 | 2.084 | 40 | 1 | 2.858 | 1.858 | 185.8 |
| 7 | 6.668 | 5.676 | 10 | 20 | 18.827 | 1.173 | 5.865 |
| 8 | 6.541 | 11.56 | 10 | 40 | 39.129 | 0.871 | 2.178 |
| 9 | 17.08 | 48.35 | 20 | 110 | 111.753 | 1.753 | 1.594 |
| 10 | 27.51 | 22.49 | 40 | 70 | 72.312 | 2.312 | 3.303 |
| 11 | 2.006 | 25.31 | 1 | 70 | 70.226 | 0.226 | 0.323 |
| 12 | 16.36 | 28.43 | 20 | 70 | 72.611 | 2.611 | 3.730 |
